# Influence of RVFV Infection on Olfactory Perception and Behavior in *Drosophila melanogaster*

**DOI:** 10.3390/pathogens12040558

**Published:** 2023-04-05

**Authors:** Stella Bergmann, Maja C. Bohn, Susann Dornbusch, Stefanie C. Becker, Michael Stern

**Affiliations:** 1 Institute for Physiology and Cell Biology, University of Veterinary Medicine Hannover, 30173 Hannover, Germany; stella.bergmann@tiho-hannover.de (S.B.); maja.charlotte.bohn@tiho-hannover.de (M.C.B.); 2 Institute for Parasitology, University of Veterinary Medicine Hannover, 30559 Hannover, Germany; susann.dornbusch@tiho-hannover.de (S.D.); stefanie.becker@tiho-hannover.de (S.C.B.)

**Keywords:** Rift Valley Fever Virus, arbovirus, neuro-immune-interaction, electroantennography, olfactory choice test

## Abstract

In blood-feeding dipterans, olfaction plays a role in finding hosts and, hence, in spreading pathogens. Several pathogens are known to alter olfactory responses and behavior in vectors. As a mosquito-borne pathogen, Rift Valley Fever Virus (RVFV) can affect humans and cause great losses in livestock. We test the influence of RVFV infection on sensory perception, olfactory choice behavior and activity on a non-biting insect, *Drosophila melanogaster*, using electroantennograms (EAG), Y-maze, and locomotor activity monitor. Flies were injected with RVFV MP12 strain. Replication of RVFV and its persistence for at least seven days was confirmed by quantitative reverse transcription-PCR (RT-qPCR). One day post injection, infected flies showed weaker EAG responses towards 1-hexanol, vinegar, and ethyl acetate. In the Y-maze, infected flies showed a significantly lower response for 1-hexanol compared to uninfected flies. At days six or seven post infection, no significant difference between infected and control flies could be found in EAG or Y-maze anymore. Activity of infected flies was reduced at both time points. We found an upregulation of the immune-response gene, nitric oxide synthase, in infected flies. An infection with RVFV is able to transiently reduce olfactory perception and attraction towards food-related odors in *Drosophila*, while effects on activity and immune effector gene expression persist. A similar effect in blood-feeding insects could affect vector competence in RVFV transmitting dipterans.

## 1. Introduction

Rift Valley Fever is a mosquito-borne zoonotic disease endemic in Africa with an expanding geographical range [1,2]. Rift Valley Fever is caused by the Rift Valley Fever Virus (RVFV), which belongs to the Phlebovirus genus in the order Bunyavirales. Mosquito-borne diseases are under constant observation as they can cause losses in livestock and contribute to mortality and long-term disability in humans with case-fatality rates ranging between 1 and 30% and disability-rate after infection between 1 and 50% [3,4]. In livestock, RVFV infection can result in high abortion rates, febrile illness, and even death in young ruminants [5,6,7]. In humans, the illness is usually mild, but some patients develop a severe form, with symptoms such as retinitis, neurological disorders, or hemorrhagic fever, which can be fatal [8,9]. RVFV was endemic in Africa and introduced into the Arabian Peninsula in 2000 [10], indicating a potential for further spread to new regions. Due to the impacts of climate change and the consequent altered geographic distribution of many insect species, vector control efforts and an understanding of the olfaction and behavior of pathogen-transmitting insects are even more urgent [11].

Many fundamental behaviors of mosquitoes and other insects are based on their ability to perceive olfactory stimuli. In infected mosquitoes, changes in host seeking and feeding behavior, as well as sensitivity to repellents, are of particular interest. The mode of processing odor stimuli can have significant implications for the transmission of vector-borne diseases [12,13,14]. This processing is dependent on internal states like sickness or starvation via neuromodulation by neurotransmitters and -peptides [15]. When infected with La Crosse virus, *Aedes* mosquitoes exhibit a higher probing frequency [16], while altered blood-feeding responses and reduced efficacy of the insect repellent N,N-Diethyl-m-toluamid (DEET) have been observed in Sindbis virus-infected mosquitoes [17]. A Dengue virus (DENV) infection has been shown to modify host-seeking and increased locomotion [18,19]. However, studies on the effects of human pathogenic viruses on mosquito behavior require high biosafety requirements, which entails a significant facility and financial effort. To mitigate these limitations, it would be advantageous to study the effects of human pathogenic viruses on non-biting insect models, such as *Drosophila melanogaster*.

As a widely used model organism for olfaction, the nervous system of Drosophila melanogaster mirrors the general olfactory organization principles of many insect species and even vertebrates [20,21]. Despite differences in the evolution of odor receptors among animals and, in our case, insect species, reflecting their respective ecology [22,23,24], odor receptor co-receptors [25,26], modulators like neuropeptide Y [27] and most higher olfactory circuit organization is well conserved across neopteran insects [28]. Furthermore, a comparison of gene orthologs enriched in the antennae reveals that the mosquito *Anopheles gambiae* shares the highest number of orthologs with *Drosophila* [29].

Moreover, *Drosophila* can also be influenced by pathogens, which can alter behavior in different ways. Cai et al. (2021) [30] showed that sensory perception could be impacted by enteropathogenic infection. Specifically, bacteria-contaminated food was avoided by the flies due to JAK/STAT signaling in ensheathing glia of the antennal lobe. Similarly, activation of IMD and Toll immunity pathways in *Escherichia coli* infected flies caused decreased oviposition through the inhibition of octopaminergic neurons by the NF-κB pathway [31]. Infection with different bacterial strains increased nitric oxide (NO) production [32]. NO is not only an effector molecule in the immune system but also an important messenger for nervous system function [33] and development [34]. NO is involved in information processing in the olfactory and visual systems, affects vesicle release at the neuromuscular junction, and plays a role in learning and memory formation [35,36,37,38,39]. These might be ways how an infection could influence perception and subsequently behavior via NO. There is limited evidence of behavioral changes due to arboviral infections in *Drosophila*. One study showed impaired climbing ability in Zika virus (ZIKV) infected flies [40]. A number of studies have been conducted on the impact of the Drosophila C virus (DCV) [41,42,43]. Sufficient concentrations of virus cause water retention and intestinal obstruction, leading to reduced activity [44], while DCV-exposed females show decreased foraging motivation towards DCV-contaminated food sources [45]. The Nora virus was also tested for influencing locomotion and in the study of Rogers et al. (2020) [46], which showed impaired geotaxis in infected flies.

Viral induced behavior changes regarding olfaction were also observed in other insect species. Infection with Baculoviruses changes the expression of specific odorant receptors in Lepidoptera causing an altered olfactory-driven behavioral response to food related odors or influencing the production of neuropeptides reducing locomotor activity and growth [47,48]. Additionally, infection state of planthoppers can alter their preferences in an olfactometer test setup regarding infected versus non-infected host plants [49].

While there are numerous studies on RVFV prevalence [50], epidemiology [51] and pathogenesis in vertebrates [52,53] or antiviral defense of insects [54,55], Lajeunsesse et al. (2020) [56] note there are no repellence tests done with RVFV. To the best of our knowledge, we are the first investigating behavioral changes in an insect in connection with a RVFV infection.

To measure the effects of RVFV, flies were injected with a dose of 1000 focus forming units (FFU) of RVFV MP12 strain. We tested the influence of an infection on electroantennograms (EAG) and olfactory choice behavior via Y-maze using different food related odors as stimuli, as well as changes in a locomotor activity monitor (LAM). RVFV replication and persistence for at least 7 days, as well as increased *NOS* expression, were confirmed by quantitative reverse transcription-PCR (RT-qPCR).

Our results show that an infection of *Drosophila* with RVFV transiently reduces olfactory perception and attraction towards food related odors, including alterations of locomotor activity. We conclude that *Drosophila melanogaster* might be a suitable model system to study mechanisms of how pathogens might influence arbovirus transmission in mosquitoes.

## 2. Materials and Methods

### 2.1. Animal Rearing

The *Drosophila melanogaster* strain *cinnabar brown* (*cnbw*) (cn[1] Cp1[ll*cnbw* 38] bw[1]/CyO; a kind gift from Jean-Luc Imler, Strasbourg) were used. Flies were reared on standard cornmeal agar (8 g agarose, 70 g cornmeal, 47 g glucose, 17 g dry yeast, 10 g sugar beet syrup, 0.08 g methyl-4-hydroxybenzoate in 800 mL water) and kept in constant darkness at 23–25 °C and 50–70% humidity.

### 2.2. Infection of Flies

All experiments were done with adult female flies. The *cnbw* fly strain had been tested negative for Wolbachia in past screenings [57]. Flies were infected at 1 to 4 days after eclosion for EAG measurements and at 2 to 3 days after eclosion for Y-maze experiments. Animals were anesthetized with CO_2_ and injected intrathoracically with 9.2 nl of either DMEM (Dulbecco′s Modified Eagle′s Medium) (Capricorn Scientific, Ebsdorfergrund, Germany) as control or 1000 FFU RVFV MP12 from a BHK (Baby Hamster Kidney) cell line in DMEM with fine glass capillaries using a Nanoject II automatic nanoliter injector (Drummond, Philadelphia, Pennsylvania, USA). Infected flies for EAG measurements were kept at ambient conditions in the laboratory (22 °C) and for Y-maze experiments at 23–25 °C and 50–70% humidity in constant darkness.

### 2.3. Electroantennography

EAG measurements were done 1 and 6 to 7 days post injection (dpi). For a continuous airflow (20.84 mL/s) and stimulus application via pulse flow (13.73 mL/s), the stimulus controller CS-55 V2 (Syntech, Ockenfels, Germany) with signal acquisition controller IDAC-2 (Syntech, Ockenfels, Germany) was used. The mixing tube between continuous flow and pulse flow contained the stimulus and was positioned 1.5 cm from the fly’s head. EAGs were recorded with glass microelectrodes pulled on a P-97 puller (Sutter Instruments, Novato, CA, USA). Capillaries were cut to a diameter of 1 μm (indifferent electrode) or 2–3 μm (measuring electrode), fire polished on a MF-830 forge (Narishige, London, UK), and filled with *Drosophila* hemolymph-like saline (modified from Stewart et al. (1994) [58]) (4.09 g/L sodium chloride (Roth, Karlsruhe, Germany), 0.37 g/L potassium chloride (Roth), 0.22 g/L calcium chloride monohydrate (Sigma-Aldrich, Schnelldorf, Germany), 4.37 g/L magnesium chloride hexahydrate (Sigma-Aldrich), 0.84 g/L sodium hydrogen carbonate (Sigma-Aldrich), 1.89 g/L HEPES (Roth), 1.89 g/L D(+)-trehalose dihydrate (Roth), 39.36 g/L sucrose (Roth), phenol red (Sigma-Aldrich)).

Animals were cold anesthetized on ice for 10 min, fixed in a cut 1000 μL pipette tip, and left to acclimatize another 10 min. Electrodes were positioned under the stereomicroscope using micromanipulators. The indifferent electrode was pricked through the ocelli triangle at the back of the head and the measuring electrode was placed on the surface of the third antennal segment.

Every stimulation series included three stimuli; each stimulus was 0.5 s long and separated by a 10 s inter stimulus interval. There was a one-minute break after every stimulation. In the first stimulation, 10 µL of apple cider vinegar (ACV) on a piece of filter paper was measured once to test the quality of electrode connection. The blank (10 µL paraffin oil (Sigma-Aldrich)) and all odor concentrations were used for three stimulations. The odor concentrations were applied in a random order. Two odor dilution series of 1-hexanol (Sigma-Aldrich) and ethyl acetate (Sigma-Aldrich) diluted in paraffin oil (Sigma-Aldrich) with 10 µL containing 1 mg, 0.1 mg, and 0.001 mg were tested on every animal. Each odor was placed on a different antenna with a 10 min break in between.

Data from EAG measurements were exported into Microsoft Excel from the EAGpro software (Syntech, Ockenfels, Germany). The averaged blank was subtracted as the threshold from every measurement value.

### 2.4. Olfactory Choice Test—Y-Maze

To evaluate the chemosensory responses, a Y-maze (MazeEngineers, Skokie, IL, USA) after Simonnet et al. (2014) [59] was used at 1 dpi and 7 dpi. In alternating positions 40 µL of the odorant or the respective solvent as control (60 µL/mL 1-hexanol in paraffin oil or ACV with water as control) was used on a piece of a cotton pad in the odor tubes. An amount of 20 cold anesthetized flies were loaded into the start vial. The assembled Y-mazes were put in varying directions into a climate chamber at 23–25 °C and 50–70% humidity in darkness for 24 h. The number of flies in the odor tubes were counted and the responsive index (RI = (number flies in odor tube—number of flies in control tube)/number of flies in both tubes) was calculated.

### 2.5. Locomotor Activity Assay

For assessing the locomotor activity of the flies at 1 dpi and 7 dpi an automated locomotor activity assay (LAM25, TriKintetics, Waltham, MA, USA) with three infrared light beams was used with 25 mm glass tubes (Roth). Tubes were prepared with approx. 4 mL of sucrose-agar (39;36 g/L sucrose, 0.15% methyl-4-hydroxybenzoate, 2% agarose in tap water) one day before the experiments. Flies were anesthetized with CO_2_ and one fly was loaded per tube in a checkerboard pattern of treatment groups into the LAM. The experiments were conducted in a climate chamber at 23–25 °C and 50–70% humidity in darkness for approx. 24 h. The data were collected and processed with the DAMSystem311 and DAMFileScan113 software (TriKinetics, Waltham, MA, USA).

### 2.6. RT-qPCR

Flies from LAM and Y-maze experiments were analyzed in pools of 10 animals, whereas flies from EAG experiments were tested individually. Samples were homogenized using steel beads in 350 µL RLT buffer (RNeasy Mini Kit, Qiagen Hilden, Germany) and 100 µL RNase-free water or sterile DMEM (Capricorn Scientific) via the TissueLyser II (Qiagen, Hilden, Germany) at 30 Hz for 30 s. The homogenates were centrifuged and total RNA was extracted from supernatant using the RNeasy Mini Kit (Qiagen) according to the manufacturer’s instructions. Eluted RNA was used in RT-qPCR.

All RT-qPCR measurements were performed in duplicates using the AriaMX real-time PCR system (Agilent Technologies Deutschland GmbH, Waldbronn, Germany).

Samples were screened for RVFV MP12 by RT-qPCR as previously published [55] and viral copies were calculated using a dilution series of a synthetic RVFV MP12-RNA standard. The assay was performed using Luna Universal Probe One-Step RT-qPCR Kit (#E3006, New England Biolabs GmbH, Frankfurt am Main, Germany) with 0.4 μM of the following primers and 0.2 μM of probe: RVFV-F (OSM_92, sense, TGA AAA TTC CTG AGA CAC ATG G), RVFL-R (OSM_93, antisense, ACT TCC TTG CAT CAT CTG ATG) and RFVLprobe (OSM_94, CAA TGT AAG GGG CCT GTG TGG ACT TGT G). Thermal profiles for all RT-qPCRs were equivalent when not stated otherwise. Reverse transcription was done at 55 °C for 10 min. Hot Start was induced at 95 °C for 1 min and amplification was set at 95 °C for 10 s and subsequently measuring fluorescence at 60 °C for 1 min for 40 cycles.

Additionally, the *dNOS* expression was determined via SYBR green PCR (Luna Universal One-Step RT-qPCR Kit, #E3005, New England Biolabs GmbH). The primers for detecting *dNOS* expression were created within a conserved sequence section in exon 16 of the NOS gene using different sequences from *Drosophila* [60,61] (Supplementary Tables S5 and S6) and the Geneious Prime 20191.1 program (Biomatter, Auckland, New Zealand): dNOS sense (GGC GAA TAA GGG ATC CCT GG) and dNOS antisense (GTA TTT TGT CGT GCG GCT CC). Identity of the PCR products was verified by sequencing using 54 ng of DNA and 100 ng/μL of primers (Microsyns SeqLab GmbH, Göttingen, Germany). The sequences were compared to the reference sequence using Geneious Prime and the pairwise alignment function (Supplementary Table S6). Fluorescence was measured at the primer specific annealing temperature of 84 °C. A melting curve from 55 to 95 °C in 0.5 °C increments was done while monitoring the fluorescence to confirm the presence of a single gene-specific amplicon at 86 °C. The expression of *dNOS* was normalized by the housekeeping gene RPL32 (TaqMan Gene Expression Assay, Dm02151827_g1, Thermo Fisher, Frankfurt am Main, Germany) using Luna Universal Probe One-Step RT-qPCR Kit (#E3006, New England Biolabs GmbH). Relative expression was determined using this formula: 2^(Cq(RPL32))^/2^(Cq(dNOS))^.

### 2.7. Statistical Analysis

Further processing and statistical analysis of the data was done with Microsoft Excel and GraphPad Prism Version 9.0.0 (GraphPad Software, San Diego, CA, USA). For comparing two groups, t-tests were conducted using the arithmetic mean and standard deviation (SD) for the EAG and Y-maze data. For the LAM data, the data of the three monitors were summarized and the average locomotor activity was calculated by the William’s mean and the SD based on the William’s mean for the *t*-tests. Levene test for equality of variances and Kolmogorov-Smirnov test for normality were conducted. For the *dNOS* fold change analysis, an outlier analysis using the Grubbs’ test was performed and the identified outlier was excluded.

## 3. Results

**Figure 1:**
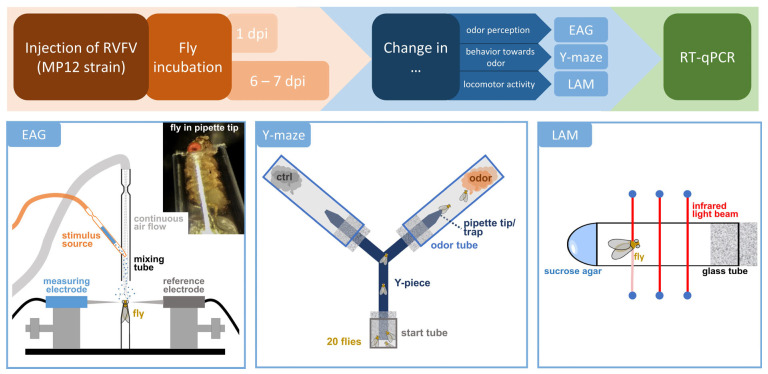
Experimental schedule. Female *cnbw* flies were injected with 1000 focus forming units (FFU) of Rift Valley Fever Virus (RVFV) and either incubated for 1 or 6 to 7 days. Changes in odor perception was measured via electroantennography (EAG). For EAG measurement, flies were fixed in a cut pipette tip. Odor stimuli were delivered in an air stream and antennal response was recorded via electrodes placed on the antenna. Changes in behavior towards odors were tested in the Y-maze. 20 flies were put into the small start tube connected via a Y-piece to the odor tubes. After 24 h, flies were counted and responsive indices (RI) determined. Changes in locomotor activity were traced with a locomotor activity monitor (LAM). Single flies were placed into a glass tube with sucrose agar. Movements were detected by three infrared light beams for 24 h. Infection status was confirmed via quantitative reverse transcription-PCR (RT-qPCR).

To elucidate infection-induced changes in perception and behavior, we injected flies with RVFV or medium (Figure 1). After 1 dpi or 6 to 7 dpi, flies were subjected to electroantennography to test for changed perception of test odors, Y-maze to test the attractive or aversive effect of test odors, and locomotor activity monitor to measure differences in the activity levels between treatment groups. Finally, infection state was assayed via RT-qPCR for RVFV and additionally expression changes of *NOS*.

### 3.1. Rift Valley Fever Virus RT-qPCR

RVFV infection of flies used in EAG, Y-maze, and LAM was verified via RT-qPCR (Supplementary Tables S1–S4). Infection was confirmed in all Y-maze and LAM samples. On 2 dpi, flies from the Y-maze experiments with 1-hexanol as test odor had a mean copy number of 3.926 × 10^6^ ± 4.668 × 10^5^ RVFV RNA copies/fly (±SEM, n = 5) and on 8 dpi of 6.706 × 10^7^ ± 9.805 × 10^6^ RVFV RNA copies/fly (±SEM, n = 5). Copy numbers of infected flies used in Y-maze with ACV as test odor were similar, with 3.567 × 10^6^ ± 2.744 × 10^5^ RVFV RNA copies/fly (±SEM, n = 5) on 2 dpi and 4.366 × 10^7^ ± 1.080 × 10^7^ RVFV RNA copies/fly (±SEM, n = 5) on 8 dpi. Copy numbers of flies from LAM experiments were also comparable, with 2.327 × 10^6^ ± 1.697 × 10^5^ RVFV RNA copies/fly (±SEM, n = 3) on 2 dpi and 2.564 × 10^7^ ± 4.277 × 10^6^ RVFV RNA copies/fly (±SEM, n = 3) on 8 dpi.

Flies from EAG were tested individually and negative tested individuals were omitted from analysis. Mean copy numbers of EAG flies on 1 dpi represent 1.720 × 10^5^ ± 4.680 × 10^4^ RVFV RNA copies/fly (±SEM, n = 10) and 2.336 × 10^7^ ± 1.137 × 10^7^ RVFV RNA copies/fly (±SEM, n = 11) on 6 to 7 dpi.

The infection with RVFV was successful (at least 92%) and a strong viral replication was observed after 1 to 2 days after injection of 1000 FFU, as well as a minor replication from 1 to 2 dpi until one week after injection.

### 3.2. Effect of RVFV on Antennal Odor Perception?

**Figure 2:**
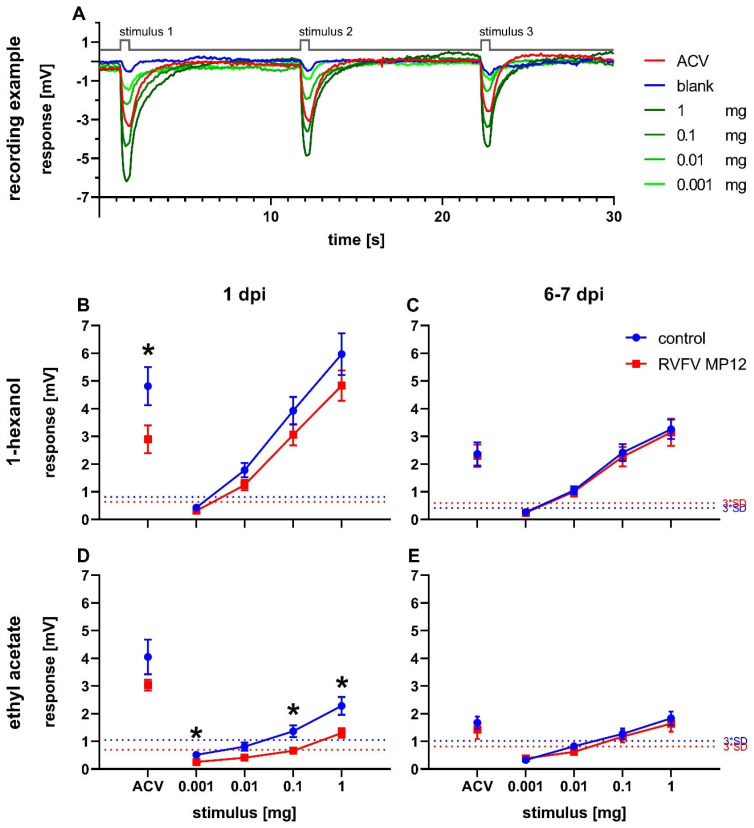
Electroantennography. Stimulus-response curves of flies infected with RVFV on 1 day post injection (dpi) or 7 dpi. (**A**) Recording example of traces from a dilution series of 1-hexanol with marked stimulus delivery of 0.5 s (grey line) with 10 s interstimulus intervals in between. (**B**–**E**) EAG measurements of responses to 1-hexanol (**B**,**C**) or ethyl acetate (**D**,**E**) and apple cider vinegar (ACV) as positive control. Mean antennal response ±SEM. Dotted lines indicate threefold SD of the response to paraffin oil (blank). (**B**) n(control) = 11; n(RVFV) = 9; (**C**) n(control) = 13; n(RVFV) = 12; (**D**) n(control) = 10; n(RVFV) = 8. (**E**) n(control) = 13; n(RVFV) = 10. Statistical analysis: multiple *t*-tests, ns ≥ 0.05, *p* < 0.05 (*).

Odor perception in flies was measured by electroantennography. The recording examples show traces from naive *Drosophila melanogaster* in response to a dilution series of 1-hexanol (Figure 2A). Stimulation resulted in a concentration-dependent deflection of the electroantennogram. In every experiment, ACV was used as a positive control and paraffin oil was used as a blank.

Antennal responses of RVFV and control flies were measured at 1 dpi and 6 to 7 dpi (Figure 2B–E). The response amplitudes were concentration-dependent for all tested odors at all tested points in time (Figure 2A–E). Infected flies showed decreased responses on 1 dpi compared to controls for both 1-hexanol and ethyl acetate, with significantly reduced responses at 0.001 mg, 0.1 mg, and 1 mg ethyl acetate (Figure 2B,D). In addition, a decrease in EAG response in infected flies was also shown for ACV at 1 dpi with a significant reduction in the 1-hexanol measurement series (Figure 2B).

At 6 to 7 dpi, a general decrease in response intensity was observed compared to 1 dpi for 1-hexanol and ACV and a minor effect for ethyl acetate (Figure 2B–E). Differences between treatments disappeared (Figure 2B–E). The higher response intensity towards 1-hexanol compared to ethyl acetate at equal concentrations at both time points was striking (Figure 2B–E).

### 3.3. Behavioral Effect of RVFV on Odor Preference

**Figure 3:**
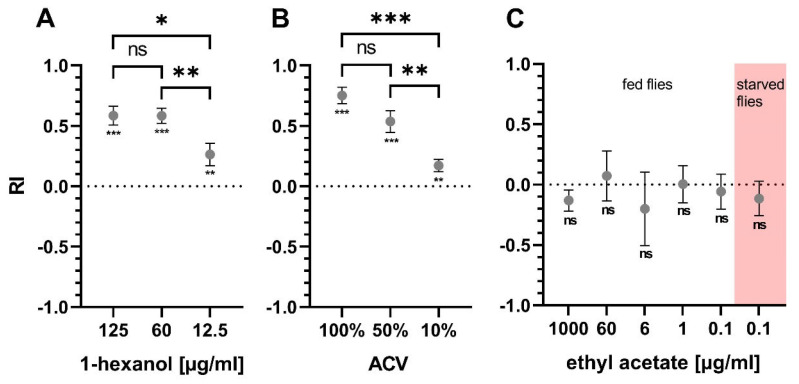
Y-maze odor attraction assay. RIs of flies confronted with different concentrations of tested compounds. RIs of naive flies tested with different concentrations of (**A**) 1-hexanol, (**B**) ACV and (**C**) ethyl acetate against diluent as control. (**A**) n(125 µg/mL) = 21; n(60 µg/mL) = 30; n(12.5 µg/mL) = 31 (**B**) n(100%; 50%; 10%) = 10 (**C**) n(1000 µg/mL) = 12; n(60; 6; 1 µg/mL) = 5; n(0.1 µg/mL fed or starved) = 15. Error bars display SEM. Statistical test: two-sample t-test (between concentrations), one-sample t-test (concentrations compared to zero), ns ≥ 0.05, *p* < 0.05 (*), *p* < 0.01 (**), *p* < 0.001 (***).

The Y-maze was used to quantify the preference of the flies towards an odor. Different concentrations of odor substances were tested in the Y-maze on untreated flies to ensure consistent results and effect sizes that allow for detection of differences between treatment groups (Figure 3). 1-hexanol was tested at three different concentrations and showed an overall attractive effect in the Y-maze (Figure 3A). The concentrations of 125 and 60 µg/mL 1-hexanol yielded high RIs above 0.5. All tested concentrations were significantly different from a theoretical value of zero. Likewise, ACV was attractive to the flies in the Y-maze setting and produced RIs above 0.5 using 100% or 50% ACV (Figure 3B). All tested ACV dilutions were significantly different from zero. Ethyl acetate was also tested in the Y-maze at five different concentrations between 1000 µg/mL and 0.1 µg/mL, but no reproducible attractive or aversive effect could be found (Figure 3C). Since ethyl acetate, like 1-hexanol and ACV, is considered a food related odor, 0.1 µg/mL ethyl acetate was also tested on flies starved for 24 h, but no change was found (Figure 3C).

**Figure 4:**
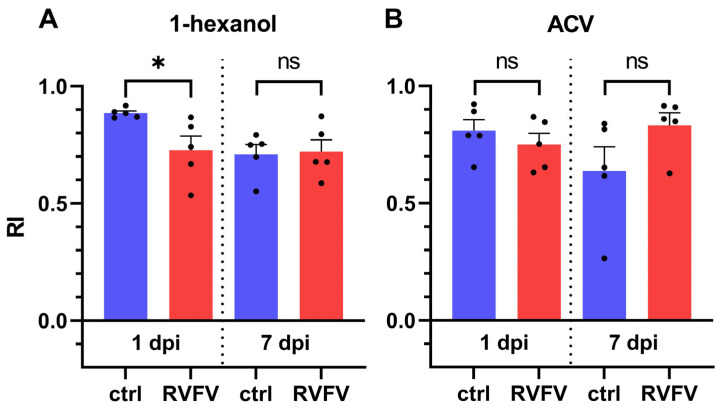
Y-maze odor attraction assay. RIs to 1-hexanol (**A**) and ACV (**B**). RIs of controls and RVFV injected flies tested with 1-hexanol (60 µg/mL) and ACV (100%) against diluent as control at 1 and 7 dpi. Shown are five biological replicates each representing the average of five Y-mazes ±SEM. Statistical test: *t*-test, ns ≥ 0.05, *p* < 0.05 (*).

After identifying effective odor concentrations, odor preferences of RVFV infected flies were compared to those of control flies in the Y-maze. A significantly lower attraction response to 1-hexanol was found in infected flies on 1 dpi (Figure 4A). Controls showed a very high attraction by 1-hexanol with a RI of 0.89 ± 0.01 (±SEM, n = 5) on 1 dpi. Even significantly decreased, RVFV infected flies were still highly attracted by 1-hexanol with a RI of 0.73 ± 0.06 (±SEM, n = 5) on 1 dpi. At 7 dpi, there was no discernible difference between responses of the treatment groups, and both still showed high attraction by 1-hexanol (ctrl: 0.72 ± 0.05 (±SEM, n = 5); RVFV: 0.71 ± 0.04 (±SEM, n = 5)). Notably, control flies showed decreased attraction towards 1-hexanol over time.

In contrast, flies showed no significant changes in RIs to ACV regarding treatment or time. RIs for ACV were also comparably high with 0.75 ± 0.05 (±SEM, n = 5) for RVFV infected flies and 0.81 ± 0.05 (±SEM, n = 5) for controls on 1 dpi. On 7 dpi infected flies had a RI of 0.64 ± 0.10 (±SEM, n = 5) and control of 0.83 ± 0.05 (±SEM, n = 5).

### 3.4. Effect of RVFV on General Locomotor Activity

**Figure 5:**
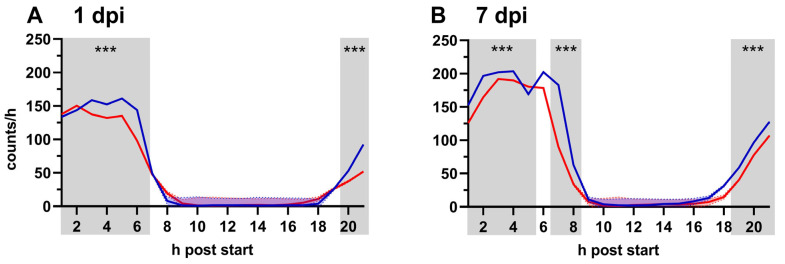
Locomotor activity at 1 dpi (**A**) and 7 dpi (**B**). Averaged (William’s mean) activity counts per hour of control (blue) and RVFV (red) infected flies ± SEM (shaded areas). At each time point, each group consists of 3 biological replicates; each replicate includes 16 flies. Grey boxes indicate significant differences between groups at individual hours post start at marked time points. Statistical analysis: *t*-tests, ns ≥ 0.05, *p* < 0.001 (***).

The locomotor activity of flies was measured under the same conditions as the Y-maze assay in constant darkness within the same daily period. Under light–dark conditions, wild type *Drosophila* exhibit a circadian activity pattern characterized by activity peaks at light onset and offset with reduced activity in the middle of the light period and lights off [62]. In general, RVFV infected flies moved significantly less than controls during the activity period (Figure 5). On 1 dpi, activity in infected flies was decreased by 11.60%; and on 7 dpi, it decreased by 17.64% compared to controls when averaged over the entire monitoring period. Notably, after a week, the activity increased within the treatment groups by 52.72% for RVFV infected flies and 63.93% for control flies averaged over the entire monitoring period with about 150 counts/h at 1 dpi and about 200 counts/h at 7 dpi at the maximum activity peak.

### 3.5. Increased Expression of dNOS in RVFV-Infected Flies

**Figure 6:**
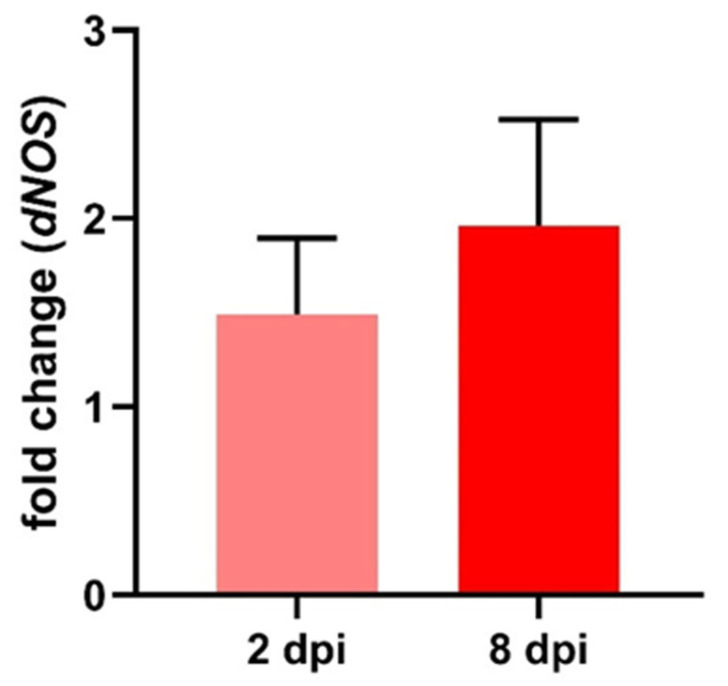
Fold change of *dNOS* expression in RVFV-injected flies normalized to controls. 2 dpi: 1.49 ± 0.41 (±SEM, n = 5); 8 dpi: 1.96 ± 0.56 (±SEM, n = 4).

Besides verifying RVFV infection, part of the Y-maze samples was also tested for relative expression of the immune-effector gene *dNOS* normalized to a housekeeping gene using RT-qPCR. When normalized to control flies, RVFV infected flies showed a small, but not significant (*t*-test), increase in *dNOS* expression with a fold change of 1.49 ± 0.41 (±SEM, n = 5) at 2 dpi and a stronger increased expression at 8 dpi of 1.96 ± 0.56 (±SEM, n = 4) fold change (Figure 6).

## 4. Discussion

### 4.1. Successful RVFV Infection and Replication

Here, we show the influence of an infection with RVFV, a mosquito-borne virus, on the olfaction and behavior of the classical model insect *Drosophila melanogaster*.

Infection with RVFV was successful and viral replication was confirmed via RT-qPCR at all tested time points. As already shown in other studies [55,63], productive infection did not lead to a higher mortality in flies in our experiments (data not shown) and correcting for dead flies in analysis was not necessary. Since flies from Y-maze and LAM experiments were tested for RVFV in pools while flies employed in EAG were tested individually due to the significance of individual flies’ data, RT-qPCR values of EAG flies tend to vary more. Nevertheless, successful infection and replication could be confirmed.

### 4.2. Altered Odor Perception

Infection with RVFV generated several changes in the response to odors and activity in infected flies compared to mock injected flies. In both infected and control flies, reproducible EAG recordings were obtained for all three tested odors (Figure 2). Ethyl acetate is an important food-related odor for *Drosophila* because it indicates the presence of *Saccharomyces* [64]. Flies are very sensitive to this odor and employ two different odorant receptors for high and low concentrations of ethyl acetate [65]. Similarly, 1-hexanol is also an important odorant for fruit flies and likewise attractive for *Culex* mosquitoes [66,67]. Its content strongly increases in ripening fruits [66] and perception of high concentrations involves up to six different odorant receptors [65]. It can be assumed that odor perception of ACV also involves multiple receptors, since ACV is a mixture of many different components. Therefore, we can explain the generally higher EAG responses to ACV and 1-hexanol compared to ethyl acetate (Figure 2B).

Infected flies showed decreased antennal responses to food-related odors at 1 dpi (Figure 2B). As for many other odorants, intensity of attractiveness or averseness seems to be correlated with summed spike activity [65]. Hence, reduced EAG activity or decreased odor perception would translate into reduced attractiveness in the olfactory choice test. In DENV infected mosquitoes, transcription of antennal genes was altered at later stages of infection and correlated with changes in odor sensitivity [18]. ZIKV has been shown to influence the sensitivity to repellents in mosquitoes [68].

We observed a significant reduction of EAG responses to ACV and ethyl acetate and a non-significant reduction to 1-hexanol (Figure 2B). On the other hand, olfactory choice for 1-hexanol in the Y-maze was significantly reduced in RVFV infected flies on 1 dpi, but not for ACV (Figure 4). Although Y-maze experiments are sensitive to various changes in ambient conditions, we considered the differences in RIs between odor concentration finding tests (Figure 3) and infection experiments (Figure 4) for the chosen concentrations to be minor. To ensure accurate comparisons, we made sure that the results being directly compared were in close temporal connection (within a few weeks). We could not test choice for ethyl acetate in the Y-maze for methodical reasons. Flies were not attracted (or repelled) by ethyl acetate over a wide range of concentrations (Figure 3C). This could be explained by the high vapor pressure of ethyl acetate as compared to the other odorants [69,70,71], which prevents its usage in the Y-maze assay. However, we consider the Y-maze superior to air-flow based olfactory choice assays because the uncomfortably small start tube enforces choice and the assay does not depend on locomotion against an air stream [72]. This is particularly important in RVFV infected flies that showed less activity.

The replication rate of RVFV was higher during the first 48 h as compared to later time points in infection (7–8 dpi). This infection kinetics could be explained by activation of various immune pathways with different delays. Some responses peak already within the first 24 h, e.g., NO-production in the JAK/STAT pathway [32,73] or antimicrobial peptide (AMP) production of the Toll and IMD pathways [74]. However, the presumably most efficient anti-viral RNAi pathway has its activity peak around 3 to 4 days [55,75], leading to control of further virus replication and pathogeny by the end of the first week after infection.

At one week post injection, no differences in odor responses between treatment groups was observed (Figure 2C). EAG responses were generally decreased for all odorants (Figure 2C). It seems possible that aging plays a greater role at this time point than infection status. Age-related decrease in olfactory response may be due to internal inflammation [30,76]. Taken together, reduced antennal responses to 1-hexanol cannot completely explain reduced responses to this odor in the olfactory choice assay.

### 4.3. Changes in Locomotor Activity

In the locomotor activity assay, *Drosophila* displayed the typical circadian activity pattern with nocturnal sleep, phases of enhanced activity in the morning and evening, and reduced, variable activity during the day [62]. In our experiments under constant darkness, flies displayed the same circadian pattern in both infected and uninfected conditions (Figure 5). In RVFV infected flies, we observed a significant reduction in activity at their active time on 1 dpi as well as 7 dpi (Figure 5). It has been reported that a ZIKV infection can reduce locomotor activity of *Drosophila* in a climbing assay [40].

However, it is very unlikely that reduced odor choice in our experiments can be explained by the reduced locomotor activity of infected flies alone, because the design of the Y-maze with its small start chamber strongly reduces possible effects of general activity [59], as opposed to, e.g., upwind-T-maze tests that strongly rely on locomotor activity [72]. Thus, in our experiments conducted for 24 h, participation rates in the test were high in both RVFV and control groups. It has been reported that a Drosophila C virus infection can decrease locomotion in infected flies by fluid imbalance leading to physical impairment due to a swollen abdomen [43,44]. In our experiments, we did not observe such apparent physical abnormalities in RVFV infected flies.

### 4.4. Insect Immune Responses to Human Pathogens

There are several principal mechanisms for a virus infection to induce changes in the nervous system and behavior: by directly affecting neurons or glia cells in the central or peripheral nervous system [77]; or indirectly via responses of the innate immune system [73] or other organs such as the gut which, in turn, affects the nervous system and behavior.

Neurotropism, as a prerequisite for direct action of a virus on the nervous system, is shown for ZIKV and DENV in mosquitos. Both viruses are able to infect neurons in cell culture, where increased spike activity was induced by ZIKV infection [77,78]. Neurotropism and neuropathology in humans are also known for these viruses [79,80,81,82]. Decreased activity in ZIKV infected mosquitoes was linked to the viral influence on the internal clock [83]. Similarly, increased locomotor activity in DENV infected mosquitoes was also ascribed to infection-based changes in the insect’s clock [84]. In *Drosophila*, influence of an infection on activity patterns and the circadian clock have been shown as well [85,86,87]. Remarkably, the rhythmicity of the circadian activity pattern in DENV infected mosquitoes was similar to that of uninfected mosquitoes [84]; this matches our observations for RVFV infected flies. Genetically disabling the circadian clock in mosquitoes reduced attraction to host odor, increased blood feeding, and altered activity patterns [88]. Kozlov et al. (2020) [89] showed that glia cells in *Drosophila* can influence the circadian clock via NO. In addition, manipulation of the thoracic ganglia, which controls the locomotion in insects—for example, via NO/cGMP signaling—cannot be excluded. RVFV, the virus used in our study, is known to cause neuropathology in humans [90] and to display neurotropism in mice [91]. In *Drosophila*, RVFV is mostly pantropic and reported to be found in a variety of different tissues including the nervous system [92,93]. Thus, RVFV induced altered olfactory perception and behavior in our experiments could have been due to direct influence of the virus on the nervous system. However, virus infection persisted at least until one week post infection, whereas altered antennal responses and olfactory choice behavior did not (Figure 2 and Figure 4).

Alternatively, indirect effects of the infection could have been mediated by the insect’s innate immune system. The main immune pathway to control viral infections is the RNAi pathway, which has, along with autophagy via the Toll-pathway, been shown to limit viral replication in RVFV infected flies [54,55]. For DENV, involvement of the Toll pathway and following AMP production [94,95,96], upregulating of the IMD pathway and its effectors [95,97], and the activation of the JAK/STAT pathway were shown [97,98]. In *Drosophila* infected with RVFV, we observed a moderate upregulation of *NOS* expression (Figure 6). Since NOS is expressed in fat body, hemocytes, and nervous system, but not in the gut or reproductive system of *Drosophila*, tissue-specific investigation of RVFV-dependent expression changes of NOS might result in even clearer results in future experiments. Upregulating of *NOS* expression and NO production, respectively, is generally associated with the JAK/STAT pathway and microbial or parasitic infection [99,100]. In *Drosophila*, NO production was increased by various bacteria strains [32], as well as increased *NOS* expression was observed in *Escherichia coli* or Plasmodium infected mosquitoes [101,102,103,104]. In locusts, NO production increases in hemocytes when challenged with bacteria [73]. NO from stimulated hemocytes can induce the second messenger cGMP in ganglionic neurons, indicating that NO is well suited as a messenger for a cross talk between immune and nervous systems [73]. Although *NOS* expression is mostly observed after microbial infection, in honeybees, upregulating of *NOS* was observed after a viral infection [105] and the JAK/STAT pathway was activated in *Drosophila* during DCV infection [106]. Additionally, low concentrations of NO are able to induce AMP production via IMD pathway [107,108]. NO and strong AMP production have cytotoxic effects with AMPs leading to apoptosis and changes in the mitochondria [76,109]. Moreover, other human viral diseases like COVID-19 also show an impairment of olfaction, presumably due to general inflammation of the respective tissue [110] and hence an immune response leading to cytotoxic effects. Multiple modes of action for the signaling molecule NO are plausible: whether it is via its function as a neurotransmitter, a direct cytotoxic effect, or secondary effects via activating following immune pathways and effectors. Nevertheless, upregulated *NOS* expression still present one week after RVFV infection in our study can explain changes in general locomotor activity but cannot completely explain the transient nature of the impairment of the observed olfactory perception and behavior. However, one week is plenty of time for compensatory mechanisms to balance the effect of a moderate increase in *NOS* expression.

### 4.5. Limitations of Our Study

A shortcoming of our study is that only one fly strain was used, but it is known that different fly strains can react differently to infections, which may affect the generalizability of our findings. Additionally, more different odors should be tested. In nature, flies and vector species are exposed to plenty of different odors, which are differently processed depending on their ecological relevance. We also only looked at the effects of a single arbovirus. Moreover, blood-feeding mosquitoes have longer adult life spans than *Drosophila*. Important phases in the life of the vector, such as blood-feeding and mating, can occur later than one week, where we did not collect data on *Drosophila*. For RT-qPCR, the viral titer was close to the detection limit for non-pooled flies, which could have affected the accuracy of the results.

## 5. Conclusions

In conclusion, quantification of electrophysiologically measured odor and behav-ioral responses in a model insect revealed a transient yet promising effect of a RVFV infection on the olfactory response and behavior of *Drosophila melanogaster*. To the best of our knowledge, this is the first study demonstrating the influence of a human pathogenic arbovirus on olfaction of *Drosophila*. With the availability of sophisticated genetic tools and the simplicity of handling in comparison to pathogen-transmitting mosquitoes that require extensive precautions in the laboratory, *Drosophila* may provide a promising platform to investigate the complex arbovirus-vector interactions. In future studies, a mechanistic investigation of various immune pathways and their influence on circadian activity rhythm and olfactory perception under viral infection would be a worthwhile prospect to explore.

## Data Availability

The PCR data presented in this study are available in the supplementary information.

## Supplementary Materials

The following supporting information can be downloaded at: https://www.mdpi.com/article/10.3390/pathogens12040558/s1, Table S1: Averaged RVFV RNA copies from flies after locomotor activity assay; Table S2: Averaged RVFV RNA copies from flies after Y-maze assay with the odor 1-hexanol; Table S3: Averaged RVFV RNA copies from flies after Y-maze assay with the odor apple cider vinegar; Table S4: Number of RVFV RNA copies from individual flies after EAG measurement; Table S5: Sequence references used for dNOS primer creation [60,61]; Table S6: Selected conserved dNOS sequence section.
