# Supplementary material for: Influence of RVFV Infection on Olfactory Perception and Behavior in Drosophila melanogaster
Source: Pathogens. 2023 Apr 5;12(4):558. doi: 10.3390/pathogens12040558 (PMC10142484; doi:10.3390/pathogens12040558)
Supplement: Supplementary file 1 [file pathogens-12-00558-s001.zip › pathogens-2280086-supplementary.pdf]

Supplements

Table S1. Averaged RVFV RNA copies from flies after locomotor activity assay.

Treatment	dpi	Replicate No.	Copies Per Fly	Average Copies Per Fly	SEM
mock	2	1	no Cq		
	2	2	no Cq		
	2	3	no Cq		
mock	8	1	no Cq		
	8	2	no Cq		
	8	3	no Cq		
RVFV	2	1	1.974×10^6	2.327×10^6	1.697×10^5
	2	2	2.694×10^6		
	2	3	2.315×10^6		
RVFV	8	1	3.585×10^7	2.564×10^7	4.277×10^6
	8	2	2.257×10^7		
	8	3	1.851×10^7		

Table S2. Averaged RVFV RNA copies from flies after Y-maze assay with the odor 1-hexanol. bdl: below detection limit.

Treatment	Dpi	Replicate No.	Copies Per Fly	Average Copies Per Fly	SEM
mock	2	1	no Cq		
	2	2	bdl		
	2	3	no Cq		
	2	4	no Cq		
	2	5	no Cq		
mock	8	1	no Cq		
	8	2	no Cq		
	8	3	no Cq		
	8	4	no Cq		
	8	5	no Cq		
RVFV	2	1	3.310×10^6	3.929×10^6	4.668 × 105
	2	2	5.823×10^6		
	2	3	3.399×10^6		
	2	4	2.879×10^6		
	2	5	4.236×10^6		
RVFV	8	1	7.271×10^7	6.706×10^7	9.805×10^6
	8	2	2.876×10^7		
	8	3	6.067×10^7		
	8	4	7.947×10^7		
	8	5	9.370×10^7		

Table S3. Averaged RVFV RNA copies from flies after Y-maze assay with the odor apple cider vinegar (ACV). bdl: below detection limit.

Treatment	Dpi	Replicate No.	Copies Per Fly	Average Copies Per Fly	SEM
mock	2	1	no Cq		
	2	2	no Cq		
	2	3	no Cq		
	2	4	no Cq		
	2	5	no Cq		
mock	8	1	no Cq		
	8	2	bdl		
	8	3	no Cq		
	8	4	no Cq		
	8	5	no Cq		
RVFV	2	1	4.630×10^6	3.567×10^6	2.744×10^5
	2	2	3.409×10^6		
	2	3	3.370×10^6		
	2	4	2.744×10^6		
	2	5	3.681×10^6		
RVFV	8	1	8.903×10^7	4.366×10^7	1.080×10^7
	8	2	3.263×10^7		
	8	3	2.319×10^7		
	8	4	2.623×10^7		
	8	5	4.723×10^7		

Table S4. Number of RVFV RNA copies from individual flies after EAG measurement.

Treatment	Dpi	Replicate No.	Copies Per Fly	Average Copies Per Fly	SEM
mock	1	1	no Cq		
	1	2	no Cq		
	1	3	no Cq		
	1	4	no Cq		
	1	5	no Cq		
mock	6	1	no Cq		
	6	2	no Cq		
	7	3	no Cq		
	6	4	no Cq		
	7	5	no Cq		
RVFV	1	1	2.442×10^{4}	1.720×10^{5}	4.680×10^{4}
	1	2	1.663×10^{4}		
	1	3	1.726×10^{5}		
	1	4	1.190×10^{5}		
	1	5	2.902×10^{5}		
	1	6	2.849×10^{4}		
	1	7	4.052×10^{5}		
	1	8	3.805×10^{5}		
	1	9	2.758×10^{5}		
	1	10	7.467×10^{3}		
RVFV	6	1	2.552×10^{3}	2.336×10^{7}	1.137×10^{7}
	6	2	1.056×10^{7}		
	7	3	1.353×10^{8}		
	7	4	1.291×10^{7}		
	6	5	4.460×10^{7}		
	6	6	4.591×10^{6}		
	6	7	3.830×10^{6}		
	7	8	8.958×10^{6}		
	7	9	1.088×10^{4}		
	7	10	3.002×10^{7}		
	7	11	6.113×10^{6}		

Table S5. Sequence references used for dNOS primer creation.

1	Sequence	*Drosophila melanogaster* nitric oxide synthase, transcript variant A (Nos), mRNA
	NCBI ref. no.	NM_078817.4
	authors	Matthews, B.B.; Dos Santos, G.; Crosby, M.A.; Emmert, D.B.; St Pierre, S.E.; Gramates, L.S.; Zhou, P.; Schroeder, A.J.; Falls, K.; Strelets, V.; Russo, S.M. and Gelbart, W.M. [60]
2	Sequence	*Drosophila melanogaster* nitric oxide synthase, transcript variant F (Nos), mRNA
	NCBI ref. no.	NM_001032069.2
	authors	Matthews, B.B.; Dos Santos, G.; Crosby, M.A.; Emmert, D.B.; St Pierre, S.E.; Gramates, L.S.; Zhou, P.; Schroeder, A.J.; Falls, K.; Strelets, V.; Russo, S.M. and Gelbart, W.M. [60]
3	Sequence	*Drosophila melanogaster* nitric oxide synthase, transcript variant K (Nos), mRNA
	NCBI ref. no.	NM_001032072.2
	authors	Matthews, B.B.; Dos Santos, G.; Crosby, M.A.; Emmert, D.B.; St Pierre, S.E.; Gramates, L.S.; Zhou, P.; Schroeder, A.J.; Falls, K.; Strelets, V.; Russo, S.M. and Gelbart, W.M. [60]
4	Sequence	*Drosophila melanogaster* nitric oxide synthase gene, complete cds
	NCBI ref. no.	AH009071.2
	utilised section	exon 16
	authors	Stasiv, Y., Regulski, M.; Kuzin, B.; Tully, T. and Enikolopov, G. [61]

Table S6. Selected conserved *dNOS* sequence section. **A** Forward sequences of the RT-qPCR amplicons with dNOS primer. Forward primer sequence labelled yellow and reverse primer sequence labelled purple. The product length is 332 bp. **B** Reference sequence from selected *dNOS* section labelled orange, consensus sequence head *D. melanogaster cnbw* labelled blue, sequence body *D. melanogaster cnbw* labelled green. Mutations are marked in yellow.

**A**	5'-GGCGAATAAGGGATCCCTGGACAGCAGTTTATCCAAGTACCACAACAAGAAGGTGCACTGCTGCAAGGC<br>GAAGGCGAAGCCCCACAATTTGACCCGTTTGAGTGAGGGAGCCAAGACAACGATGCTGCTGGAGATCTGTG<br>CACCTGGCTTGGAGTACGAGCCGGGTGATCATGTGGGCATCTTTCCGGCGAATCGAACGGAACTGGTCGAC<br>GGACTGCTAAATCGACTGGTGGGTGTGGATAATCCCGACGAGGTGCTGCAGTTGCAATTGCTAAAGGAAAA<br>GCAGACATCGAATGGTATATTCAAGTGCTGGGAGCCGCACGACAAAATAC-3'
**B**	GGCGAAGCCCCACAATTTGACCCGTTTGAGTGAGGGAGCCAAGACAACGATGCTGCTGGAGATCTGTGCACC<br>GGCGAAGCCCCACAATTTGACCCGTTTGAGTGAGGGAGCCAAGACAACGATGCTGCTGGAGATCTGTGCACC<br>GGCGAAGCCCCACAATTTGACCCGTTTGAGTGAGGGAGCCAAGACAACGATGCTGCTGGAGATCTGTGCRCC<br><br>TGGCTTGGAGTACGAGCCGGGTGATCATGTGGGCATCTTTCCGGCGAATCGAACGGAACTGGTCGACGGACT<br>TGGCTTGGAGTACGAGCCGGGTGATCATGTGGGCATCTTTCCGGCGAATCGAACGGAACTGGTCGACGGACT<br>TGGCTTGGAGTACGAGCCRGGTGATCATGTGGGCATCTTTCCGGCGAATCGAACGGAAYTGGTCGACGGACT<br><br>GCTAAATCGACTGGTGGGTGTGGATAATCCCGACGAGGTGCTGCAGTTGCAATTGCTAAAGGAAAAGCAGAC<br>GCTAAATCGACTGGTGGGTGTGGATAATCCCGACGAGGTGCTGCAGTTGCAATTGCTAAAGGAAAAGCAGAC<br>GCTAAATCGACTGRTGGGTGTGGATAATCCCGACGAGGTGCTGCAGTTGCAATTGCTAAAGGAAAAGCAGAC<br><br>ATCGAATGGTATATTCAAGTGCTGGGAGCCGCACGACAAAAT<br>ATCGAATGGTATATTCAAGTGCTGGGAGCCGCACGACAAAAT<br>ATCGAATGGTATATTCAAGTGCTGGGAGCCGCACGACAAAAT
